# Electrochemical Synthesis, Magnetic and Optical Characterisation of FePd Dense and Mesoporous Nanowires

**DOI:** 10.3390/nano13030403

**Published:** 2023-01-19

**Authors:** Deepti Raj, Gabriele Barrera, Federico Scaglione, Federica Celegato, Matteo Cialone, Marco Coïsson, Paola Tiberto, Jordi Sort, Paola Rizzi, Eva Pellicer

**Affiliations:** 1 Dipartimento di Chimica e Centro Interdipartimentale NIS (Nanostructured Interfaces and Surfaces), Università di Torino, Via Pietro Giuria 7, 10125 Torino, Italy; 2 Istituto Nazionale di Ricerca Metrologica (INRIM), Str. delle Cacce 91, 10135 Torino, Italy; 3 Physics Department, University of Genova, Via Dodecaneso 33, 16146 Genova, Italy; 4 Universitat Autònoma de Barcelona, Campus de la UAB, Bellaterra, 08193 Barcelona, Spain; 5 Institució Catalana de Recerca i Estudis Avançats (ICREA), Pg. Lluís Companys 23, 08010 Barcelona, Spain

**Keywords:** nanowires, FePd, mesoporous, electrodeposition, SERS

## Abstract

Dense and mesoporous FePd nanowires (NWs) with 45 to 60 at.% Pd content were successfully fabricated by template- and micelle-assisted pulsed potentiostatic electrodeposition using nanoporous anodic alumina and polycarbonate templates of varying pore sizes. An FePd electrolyte was utilized for obtaining dense NWs while a block copolymer, P-123, was added to this electrolyte as the micelle-forming surfactant to produce mesoporous NWs. The structural and magnetic properties of the NWs were investigated by electron microscopy, X-ray diffraction, and vibrating sample magnetometry. The as-prepared NWs were single phase with a face-centered cubic structure exhibiting 3.1 µm to 7.1 µm of length. Mesoporous NWs revealed a core-shell structure where the porosity was only witnessed in the internal volume of the NW while the outer surface remained non-porous. Magnetic measurements revealed that the samples displayed a soft ferromagnetic behavior that depended on the shape anisotropy and the interwire dipolar interactions. The mesoporous core and dense shell structure of the NWs were seen to be slightly affecting the magnetic properties. Moreover, mesoporous NWs performed excellently as SERS substrates for the detection of 4,4′-bipyridine, showing a low detection limit of 10^−12^ M. The signal enhancement can be attributed to the mesoporous morphology as well as the close proximity of the embedded NWs being conducive to localized surface plasmon resonance.

## 1. Introduction

Metallic nanostructures hold promising potential for applications in different fields, such as nanoactuators, sensing, catalysis, etc. [1]. Besides, mesoporous metallic nanostructures have been increasingly showcasing their capability in an abundance of applications including voltage-control of magnetism, electrocatalysis, sensing, etc., owing to their porous architecture (pore sizes in the range of 2 nm to 50 nm), high thermal and electrical conductance, and other interesting properties [2,3,4]. In the same thread, dense and mesoporous nanowires (NWs) possess an array of applications credited to their elongated shape and anisotropic physical and magnetic properties, such as in biomedicine, biotechnology, ultrahigh-density magnetic storage, storage media applications, electrocatalysis, surface-enhanced Raman spectroscopy and microelectromechanical systems (MEMS) [5,6,7,8]. Specifically, bimetallic alloys with a magnetic transition element (Fe, Co, Ni) have garnered immense attention for their multifaceted nature [9,10]. FePd, being one such bimetallic alloy, holds excellent promise where properties of the transition metal (Fe) merge with that of the noble metal (Pd) [11,12,13]. The intrinsic magnetic properties of Fe can be easily exploited in an FePd alloy. This alloy also possesses enhanced catalytic efficiency among other applicatory capabilities [14]. There have been numerous applications displayed by FePd alloys, e.g., hydrogen separation, environmental remediation, membrane hydrogenation reactions, sensors and actuators [15], magnetic drug delivery [16], and catalysis [17,18]. They have also garnered extensive attention attributed to their extraordinary magnetostrictive and shape memory effects [19]. Besides, FePd alloy can be dealloyed by readily dissolving Fe, leading to the formation of a nanostructured Pd-rich material with its own unique properties (such as a large surface area to volume ratio) [10,20].

For the fabrication of NWs, electrodeposition within nanoporous templates is an impressively proficient method [15]. Not only it is a cost-effective and high throughput synthesis technique, but it also allows for fine-tuning of the attributes of the target sample, such as chemical composition, and physical and mechanical properties, by simply and conveniently altering the deposition parameters [21]. For co-depositing two metals, their respective reduction potentials should be (nearly) the same [22]. But that is not the case for co-depositing Fe and Pd as both have a large difference in their reduction potentials, which makes it challenging. Thus, the FePd electrodeposition essentially requires a suitable complexing agent that would shift the reduction potential of Pd towards the less-noble Fe while keeping the electrolyte chemically stable [22,23,24]. In addition, hydrogen evolution takes place during the process as a side reaction [15]. The generated gas bubbles might block the pores and thus interrupt the metal deposition [15]. Hence, to avoid this from happening, the pulse technique is employed, which has been shown to minimize the blockage of pores [15]. Furthermore, the formation of a high number of OH^-^ ions within the pores as a result of hydrogen evolution leads to facile precipitation of hydroxides, in particular Fe(OH)_3_ [15]. To prevent this, 5-sulfosalicylic acid is added, which forms a stable Fe^3+^ complex precluding hydroxide formation [15]. The pulse plating technique also decreases the occurrence of concentration gradients and keeps the pH constant. During the off-time, no metal deposition takes place. This allows for the balancing of concentration gradients of metal ions as well as the rise of hydrogen bubbles [25]. Notably, dense Fe-Pd NWs have been previously electrodeposited but no studies have focussed on fabricating their mesoporous counterparts, to the best of our knowledge [19,22,26,27,28,29].

When it comes to the synthesis of mesoporous metallic nanostructures, typical processes include soft-templating, hard-templating, and solution-phase approaches [30]. Specifically, soft templates such as micelle assemblies have come to the forefront in recent years. Micelle assemblies were first brought to light by Yamauchi and co-workers for the electrodeposition of Pt metal from diluted (1.0 wt.%) non-ionic surfactant solutions [31]. This procedure has also been extended to bimetallic systems [30]. Among all soft-templating methods, electrochemical deposition is the most suitable and straight-forward technique to reduce the metal ions in a solution [30]. So far, commercial amphiphilic polymers like P-123, and F-127 of Brij-58, have been used as common non-ionic surfactant assemblies above their critical micelle concentration (cmc) [30]. P-123 forms micelles in acidic water above its critical micelle concentration, with the core made of poly(propylene oxide) (PPO) block and the shell of poly(ethylene oxide) (PEO) units. During the electrodeposition process, P-123 micelle assemblies gather spontaneously at the solid–liquid interface, and the cationic species (e.g., Pd(II)) can coordinate with the shell of these micelle self-assemblies [32]. In this way, porosity can be generated in the electrodeposited NWs, which is critically dependent on the extent and strength of this coordination [30].

Surface-enhanced Raman scattering (SERS) is a well-known and remarkable sensing technique that facilitates trace-level molecular detection and also finds a vast range of usage in analytical applications, such as in catalysis, biology, biomedicine, materials science, food science, art conservation, environmental analysis, etc. [33,34]. SERS utilizes a nanostructured metallic substrate, which adsorbs the analyte molecules and amplifies the Raman signals by factors up to 10^8^ or even larger. Both electromagnetic and chemical enhancements have been attributed as the reason behind SERS enhancement, but the former is more widely accepted [35]. This is based on the phenomenon of localized surface-plasmon resonance, LSPR, which involves the excitement of the surface-electron oscillations caused by resonant coupling of the incident light and surface-plasmons of the substrate, resulting in overall signal enhancement [36,37,38]. It also encompasses the generation of ‘hotspots’ which are regions of highly intense electric field The origin of chemical enhancement lies in the resonant effect arising from the analyte-substrate charge-transfer complex [39]. Although Au and Ag-based substrates have been established as the best for SERS, it is still crucial to develop alternative SERS-active substrates to make this technique more cost-effective, precise, and sustainable [40].

In this paper, we present the successful fabrication of a series of dense and mesoporous FePd NWs with different compositions and diameters. The structural and magnetic properties of the obtained NWs were investigated. Mesoporous NWs were tested as SERS-active substrates and an excellent performance was recorded.

## 2. Materials and Methods

The FePd NWs with varying compositions and diameters, both dense and mesoporous, were fabricated by pulsed potentiostatic template-assisted electrodeposition. Commercial Anodisc 25 (Whatman, Cytiva, Marlborough, MA, USA) was used as the Anodised Aluminium Oxide (AAO) template discs with a diameter of 25 mm, membrane thickness of 60 µm, and average pore sizes of 20 nm and 100 nm. Additionally, commercially available Polycarbonate (PC) (Whatman, Cytiva, Marlborough, MA, USA) templates of pore size 30, 50, and 100 nm were used. These membranes act as hard/soft templates for the growth of the NWs. A 30 nm thick Au layer was sputter-deposited onto the backside of the membranes using an AJA international sputtering system (AJA International, Inc. Scituate, MA, USA) under an argon atmosphere to provide electrical contact and serve as working electrodes for electrodeposition.

To synthesize the dense FePd NWs, an aqueous electrolyte containing 0.01 M Tetraamminepalladium(II) chloride Pd(NH_3_)_4_Cl_2_ (Merck KGaA, Darmstadt, Germany), 0.05 M Iron(III) sulfate hydrate Fe_2_(SO_4_)_3_xH_2_O (Merck KGaA, Darmstadt, Germany), 0.06 M 5-Sulfosalicylic acid dihydrate (SSA) C_7_H_6_O_6_S·2H_2_O (Merck KGaA, Darmstadt, Germany), and 0.3 M Ammonium sulfate (NH_4_)_2_SO_4_ (Merck KGaA, Darmstadt, Germany) was used. This electrolyte was taken from earlier publications [23,41,42] and will be referred to as the FePd electrolyte in future mentions. For the preparation of mesoporous NWs, a micelle-forming surfactant was added to the aqueous FePd electrolyte, i.e., P123 triblock co-polymer (Merck KGaA, Darmstadt, Germany), which induces porosity by its micellar action. First, it was dissolved in the Pd(NH_3_)_4_Cl_2_ (1:1 ratio, e.g., 15 mg P-123 in 15 mL Pd(NH_3_)_4_Cl_2_) and stirred at room temperature for two days [30]. Afterwards, when P123 was completely dissolved, 0.05 M Fe_2_(SO_4_)_3_xH_2_O, 0.06 M SSA, and 0.3 M (NH_4_)_2_SO_4_ were added to this (Pd(NH_3_)_4_Cl_2_ + P123) aqueous solution. This electrolyte will be referred to as FePd+P123 in subsequent sections of this paper. The pH of both the aqueous electrolytes was adjusted to 5.0 for all experiments using ammonium hydroxide. All the solutions were prepared from reagents grade chemicals and de-ionized water.

All the electrodeposition experiments were carried out at room temperature under stagnant conditions. A typical three-electrode setup was used—a Pt spiral as counter electrode and a saturated Ag/AgCl (3 M) reference electrode. The metalized membranes were placed vertically within the electrolyte using a custom-made holder. All electrode potentials were in reference to the potential of the Ag/AgCl electrode. Electrodeposition was carried out in pulsed potential mode using PGSTAT302N Autolab potentiostat/galvanostat (Metrohm-Autolab B. V. Utrecht, The Netherlands). Two alternating potential steps were applied—the deposition potential E_on_ and the resting potential E_off_. The duration for which E_on_ was applied was termed here as t_on_, which was set at 30 s, while that for E_off_ was t_off_ at 90 s. The whole electrodeposition was repeated 60 times for 2 h. Here, E_on_ was varied from −1.05 V to −1.3 V to obtain NWs with different compositions while E_off_ was kept constant at −0.46 V vs. Ag/AgCl. A representative schematic diagram of pulsed potentiostatic electrodeposition is provided in Figure S1 of the Supplementary File. Following electrodeposition, the samples were repeatedly rinsed with de-ionized water. To release the NWs, the AAO template was dissolved in 10% *w/w* NaOH solution at 80 °C for 10 min while the PC template was dissolved in CHCl_3_ at room temperature for a few minutes. The supernatant was then removed and the freed NWs were washed several times in distilled water. Afterward, the NWs were kept dispersed in ethanol and sonicated for a few minutes.

The evolution of the morphology and the stoichiometry was studied via a Zeiss Merlin field-emission scanning electron microscopy (FESEM) (Zeiss Group, Oberkochen, Germany) equipped with an energy-dispersive X-ray spectrometer (EDS) (Oxford Instruments, Abingdon, UK) and a JEOL JEM-2011 High-resolution transmission electron microscope (HRTEM) (JEOL Ltd., Tokyo, Japan) operated at 200 kV. To observe them under FESEM, a small blob of ethanol containing the dispersed NWs was dropped onto the carbon tape covering a FESEM stub. The structure of the samples was examined with a Panalytical X-pert X-ray Diffractometer in Bragg–Brentano geometry (Panalytical, Almelo, The Netherlands) with monochromatic Cu K_α_ radiation.

Room-temperature magnetic measurements were performed using a vibrating sample magnetometer (VSM) (Lake Shore Cryotronics Inc., Westerville, OH, USA) operating with an applied magnetic field (H) in the range ±15 kOe. The hysteresis curves were obtained by applying H along the direction parallel (PA) and perpendicular (PE) to the major axis of the NWs. The dia-/para-magnetic signal of the sample-holder and the porous membranes was duly taken into account and properly subtracted.

SERS measurements were carried out using a Renishaw inVia Raman Microscope (Renishaw, Wotton-under-Edge, England) with 785 nm laser line (50% power, 20 × ULWD objective, 10 acquisitions with acquisition time of 40 s each), and 4,4′-bipyridine (bipy) as the probe molecule. The NWs were kept embedded in the template and subjected to a 6-min treatment in 10% *w/w* NaOH at 80 °C to partially dissolve the template and expose the NWs for the SERS measurement. Then they were rinsed with distilled water and air-dried. Freshly prepared ethanol solutions of bipy of low concentrations were utilized namely, 10^−10^ M and 10^−12^ M. The NWs were incubated in each solution for 20 min followed by air-drying. Finally, the measurements were performed.

## 3. Results and Discussion

### 3.1. Dense Nanowires

**Figure 1:**
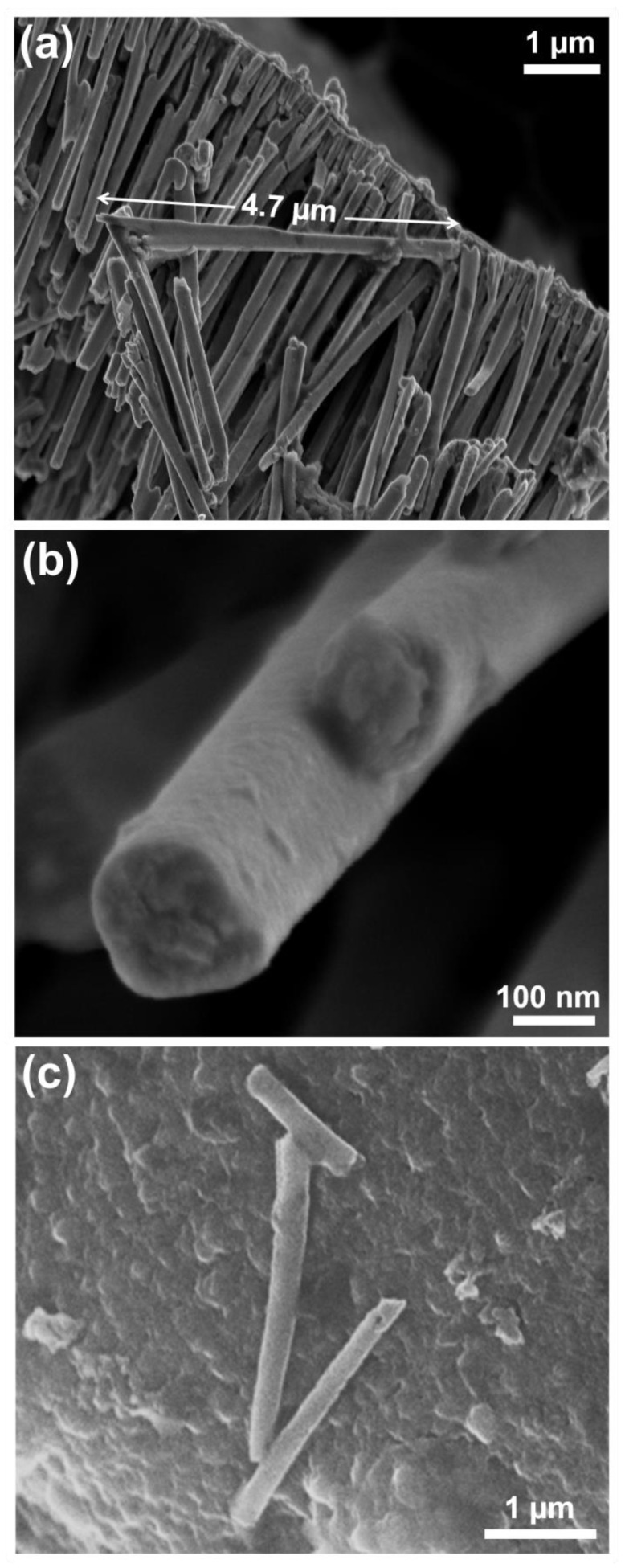
FESEM images of FePd NWs obtained by electrodeposition at E_on_ = −1.2 V on AAO membranes of (**a**,**b**) 20 nm and (**c**) 100 nm in diameter.

Dense and long NWs of different diameters and compositions were prepared using the FePd electrolyte with varying deposition potentials. Figure 1 exhibits the FESEM images of dense NWs obtained from electrodepositing at −1.2 V vs. Ag/AgCl over Au sputtered-AAO membranes of pore sizes of 20 and 100 nm. In Figure 1a, an array of NWs attached to the Au layer can be observed, showing the typical branched morphology at the top inherited from the AAO membrane. They are continuous with slightly different lengths, the longest being 4.7 µm. The mean diameter was calculated to be 157 nm with a standard deviation of 22 nm. As evidenced in Figure 1b, a magnified look at a single NW proves that these NWs are clearly dense. Remarkably, the NW diameters did not match the nominal pore size. This observation has been previously reported for Anodisc^TM^ membranes from Whatman. The difference was particularly large for the 20 nm pore-size membranes [43,44,45]. Figure 1c shows a couple of dense NWs obtained at the same E_on_ value on an AAO membrane of 100 nm in diameter, featuring a composition of Fe_56_Pd_44_; hence, similar to the previous case. The flat surface on which these NWs lay was the carbon tape used to observe them under the FESEM.

**Figure 2:**
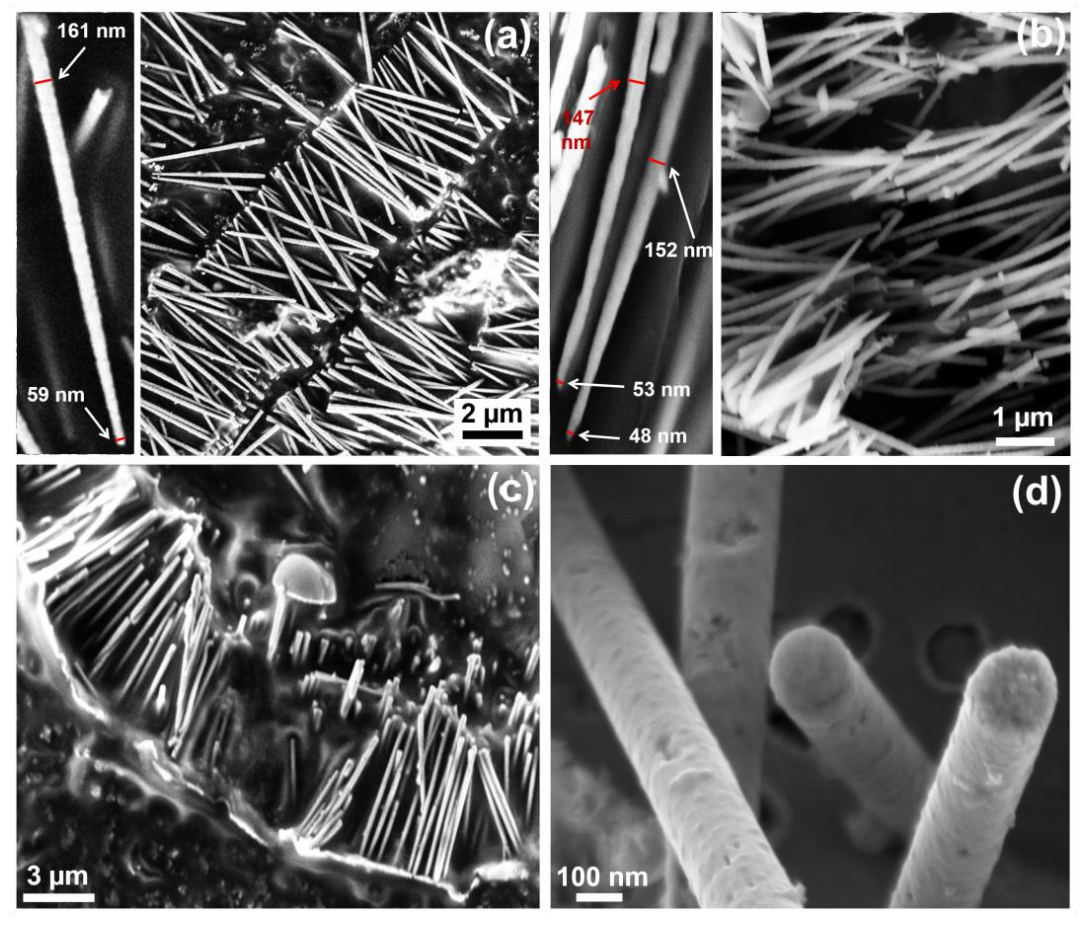
FESEM images of FePd NWs obtained by electrodeposition on PC membrane at E_on_ of (**a**) −1.05 V, (**b**) −1.15 V, (**c**) −1.2 V, and (**d**) −1.3 V.

For the sake of comparison, another set of dense NWs was obtained using the PC membrane for electrodeposition. The electrodepositing electrolyte was the same, i.e., FePd. Figure 2 assembles all the dense NWs obtained from electrodepositing over PC membranes at different deposition potentials. Figure 2a shows the FESEM image of dense NWs produced at E_on_ = −1.05 V attached to the Au layer. The composition, as determined by EDS, is Fe_55_Pd_45_. The NWs are cylindrical in shape, with a rough texture on the outer surface, as shown in the inset of Figure 2a. The NW in the same inset has a small diameter of 59 nm at the tip that becomes gradually larger throughout the length, going up to 161 nm. This cigar-like shaped morphology on NWs grown on PC templates has been previously described [46]. Figure 2b shows the FESEM image of NWs obtained at E_on_ = −1.15 V where the NWs were attached to the Au layer, after dissolving the template. The chemical composition of the NWs remained virtually the same (Fe_51_Pd_49_), and was similar to the previous value found on NWs grown on AAO templates (Fe_47_Pd_53_) (see Table 1). The longest NW holds a length of 4.6 µm. Similarly, these NWs possess a cylindrical shape but a smoother surface texture, visible in the inset of Figure 2b. The two NWs displayed in this inset have a small diameter at the tip of ~51 nm, while a wider diameter of 149 nm on average is determined for this sample. The FESEM images of NWs electrodeposited at E_on_ of −1.2 V and −1.3 V on a PC membrane of 100 nominal pore size are depicted in Figure 2c,d, respectively. The achieved compositions were Fe_55_Pd_45_ and Fe_60_Pd_40_, suggesting a slight enrichment in Fe upon making the applied potential more negative (Table 1). This observation has also been made by other authors when depositing FePd NWs from a ammonium-sulfosalicyclic electrolyte [25]. The longest NWs were 5.2 and 7.1 µm in length, respectively.

### 3.2. Mesoporous Nanowires

**Figure 3:**
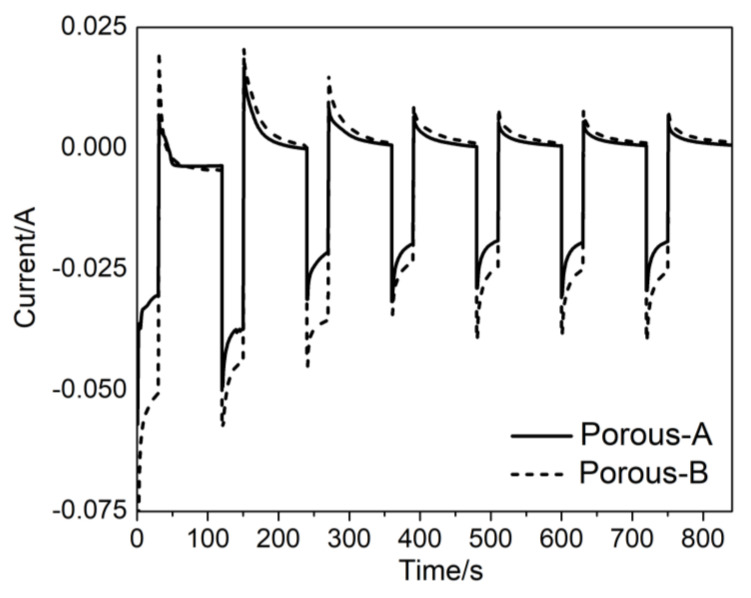
Pulsed potentiostatic transients recorded during the deposition of FePd NWs on AAO templates of 20 and 100 nm in diameter, both for E_on_ = −1.2 V.

Along with the dense NWs, mesoporous counterparts have also been produced by electrodepositing from the FePd + P123 electrolyte at E_on_ = −1.2 V vs. Ag/AgCl over 20 and 100 nm Au-sputtered AAO templates; from here onwards the former will be addressed as Porous-A while the latter as Porous-B samples. Figure 3 displays the pulsed potentiostatic transients for Porous-A and Porous-B where current vs. time is recorded. The electrodeposition in both cases appeared to be homogeneous and consistent.

**Figure 4:**
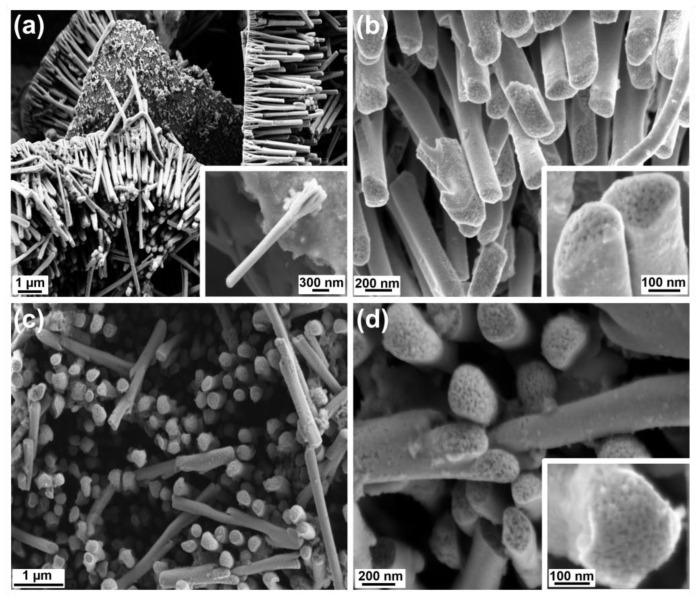
SEM images of Fe_40_Pd_60_ mesoporous NWs: (**a**) Porous-A, inset shows a single free NW at higher magnification; (**b**) magnified view of Porous-A; (**c**) Porous-B; (**d**) magnification of Porous-B; and insets of (**b**,**d**) give a closer look at the mesoporosity.

**Figure 5:**
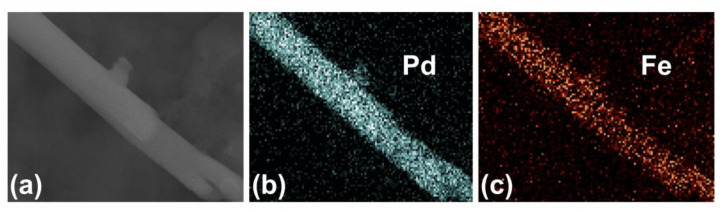
EDS maps of Porous-B NW (**a**) showing the presence and distribution of Pd (**b**) and Fe (**c**) throughout the length of the NW.

Figure 4a,b display the FESEM images of the first kind, i.e., Porous-A. In Figure 4a the collection of FePd NWs can be seen attached to the Au layer. In the inset, a free NW can be viewed in a well-defined way. It has the characteristic branched structure formed as a result of the AAO membrane. The surface is regular and smooth. No mesoporosity could be observed at this level. But as we increased the magnification further, as showcased in Figure 4b, a clear mesoporosity was evidenced at the top of the NW. Interestingly enough, this mesoporosity was only present throughout the inside the NW while the outer part was dense. This essentially formed a core-shell structure. The inset of Figure 4b gives an even better look at the top of the NW, highlighting the mesoporosity. The composition was determined to be Fe_40_Pd_60_, slightly enriched in Pd as compared to the composition previously determined for dense counterparts obtained at the same E_on._ For the Porous-B sample, similar observations have been made. In Figure 4c the top view of the NW array is apparent which in Figure 4d is visible at a higher magnification and in a better clarity. The inset of Figure 4d further reveals the presence of mesoporosity throughout the inside of the NW. For this sample too, the core-shell structure is obvious. At this stage, it is not clear yet why mesoporosity did not span the entire NW. We hypothesize that the micelles do not efficiently interact with the surface of AAO channels and a continuous, non-porous shell forms. Meanwhile, micelles could be efficiently adsorbed on the as-grown dense FePd shell and mesoporosity would then develop. Figure 5 shows the EDS maps of a Porous-B NW, establishing the presence of Pd and Fe homogeneously spread throughout the NW, so suggesting the presence of a single phase formed during the synthesis. No oxidation is observed and the overall composition of the NWs comes out to be Fe_40_Pd_60_. Table 1 summarizes the similarities and differences between Porous-A and Porous-B samples.

**Figure 6:**
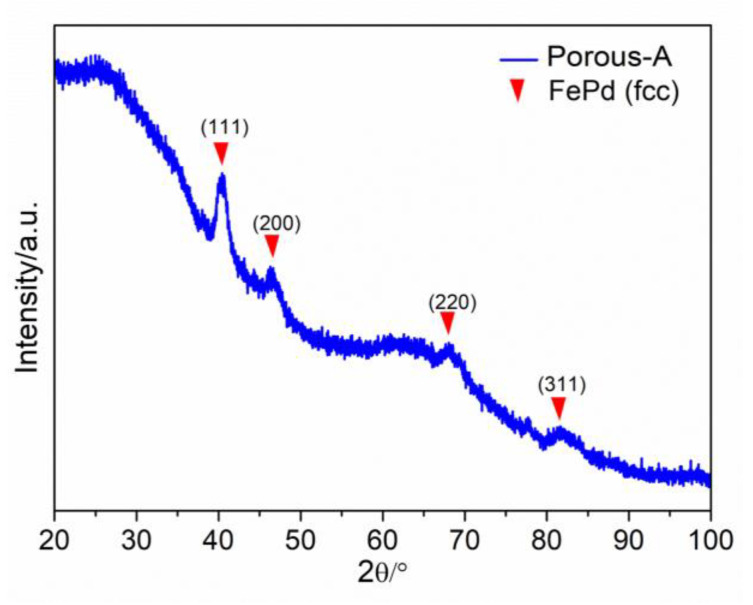
XRD pattern of the as-deposited Porous-A embedded in the AAO template.

From the Fe-Pd phase diagram, it is apparent that the Fe_40_Pd_60_ alloy in stable conditions must be constituted by two phases: FePd and FePd_3_. Nevertheless, the EDS map (Figure 5) shows a homogeneous distribution of Fe and Pd atoms along the sample, indicating the formation of just one phase with composition Fe_40_Pd_60_. To determine the structure of the phase observed by the EDS map, XRD was performed on the as-deposited Porous-A NW array and is reported in Figure 6. The NWs were kept embedded in their AAO template for obtaining the XRD pattern. Reflections related to an fcc FePd crystalline phase were detected and no secondary phases were present. This result confirmed the EDS observation and showed that the use of electrodeposition can promote the crystallization of an FePd phase rich in Pd by hindering the formation of FePd_3_. From a rough estimation of the lattice parameter for Porous-A, a_0_ comes out to be 0.388 nm, which is in accordance with the a_0_ of 0.386 nm for the FePd phase reported in the literature and related to a phase of composition Fe_50_Pd_50_; the difference in lattice parameter observed for Porous-A sample was due to a change in composition from Fe_50_Pd_50_ to Fe_40_Pd_60_ [47,48].

**Figure 7:**
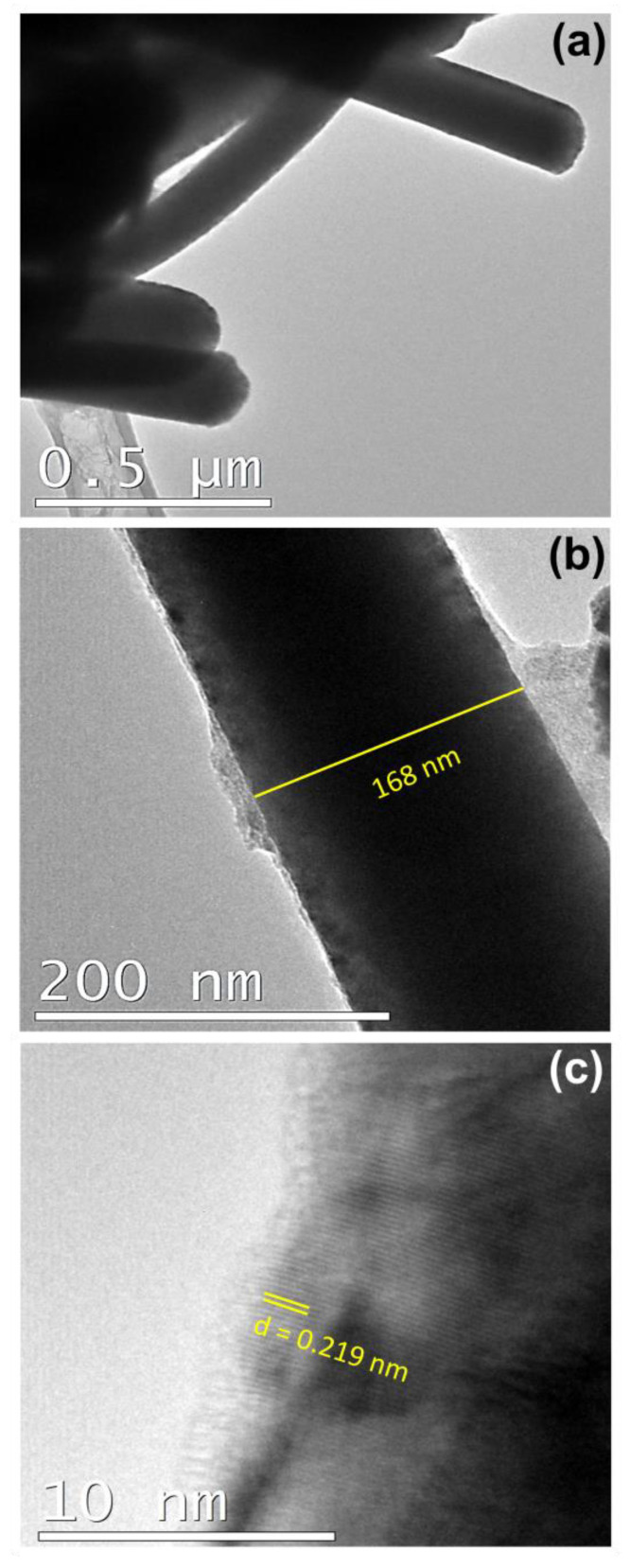
Bright-field TEM images of Porous-A NWs: (**a**) A bunch of NWs, (**b**) diameter of a single NW and (**c**) close-up view of the wall.

The morphology of the NWs was also observed by TEM after completely dissolving the template, as described previously in the case of the FESEM sample preparation. A very small amount of ethanol containing the dispersed NWs was dropped onto a Cu grid to be observed under TEM. Figure 7 shows the bright-field TEM image of the as-deposited mesoporous FePd NWs, Porous-A. From the high-resolution (HR) bright-field TEM image in Figure 7c, the sharply defined lattice planes with distances of 0.219 nm can be observed. This calculated lattice space was in accordance with that obtained from XRD measurement corresponding to the (111) plane of the FePd phase. Note that the inner mesoporosity of the NWs could not be disclosed by TEM because of both the relatively large diameter of the NWs and the occurrence of a non-porous shell.

### 3.3. Magnetic Studies

**Figure 8:**
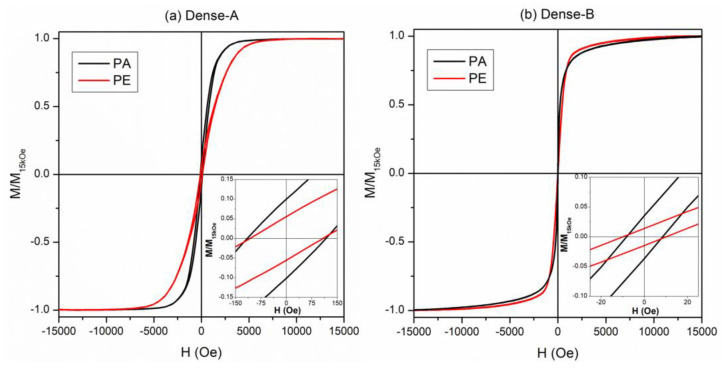
Room-temperature hysteresis loops of (**a**) Dense-A and (**b**) Dense-B NW arrays by applying H along the direction parallel (PA) and perpendicular (PE) to the major axis of the NWs. Inset: enlargement of hysteresis loops at a low magnetic field.

All the investigated NWs were ferromagnetic despite their relatively large Pd content. Ferromagnetic properties in Fe_x_Pd_1−x_ alloys are well documented, even for Pd percentages exceeding 80 at.% [49]. The room-temperature hysteresis loops of the Dense-A and Dense-B samples are shown in Figure 8. The curves obtained by applying H along the direction parallel (PA) and perpendicular (PE) to the major axis of the NWs were compared for both samples; all curves were normalized to the magnetization value at H = 15 kOe. The values of the magnetic properties such as saturating field (H_s_), coercive field (H_c_), magnetic susceptibility evaluated at the coercive field (χ_Hc_), and the squareness ratio (M_r_/M_s_) are reported in Table 2.

Both samples were ferromagnetic with well-defined hysteretic behavior. In the Dense-A NW array, the hysteresis loops measured along the PA and PE direction showed differences in the magnetic behavior (see Figure 8a), indicating an anisotropic behavior of this sample. In particular, the PA magnetization approaches the saturation at lower H_s_, with a reversal mechanism over a narrower field interval with respect to PE one. The coercive field slightly decreases from the PA to PE direction. These results suggest that the PA direction, i.e., along the major axis of the NWs, is the easy axis of magnetization. Conversely, the hysteresis loops of the Dense-B NW array sample measured along the PA and PE directions appeared almost superimposed, suggesting an almost isotropic magnetic behavior (see Figure 8b). A slight preference for the PA direction as the easy axis of magnetization can be inferred from the inset of Figure 8b and the χ_Hc_ values (see Table 2). However, both curves display a reversal of magnetization characterized by a steep jump with a χ_Hc_ higher than that of the Dense-A sample. Moreover, after the magnetization field jump, a slow approach to saturation ending at H ≈ 15 kOe is observed indicating a higher difficulty of the magnetic moments to reach the complete alignment with the applied magnetic field. The coercivity values were coincident for both PA and PE directions in the Dense-B sample but resulted in them being one order of magnitude lower than that measured in the Dense-A sample.

**Figure 9:**
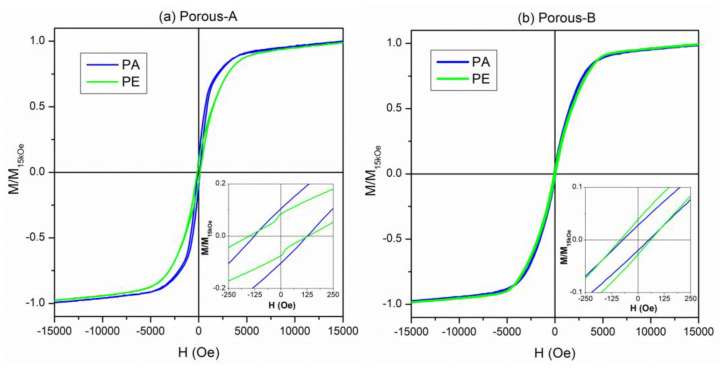
Room-temperature hysteresis loops of (**a**) Porous-A sample and (**b**) Porous-B sample by applying H along the direction parallel (PA) and perpendicular (PE) to the major axis of the NWs. Inset: enlargement of hysteresis loops at a low magnetic field.

The room-temperature hysteresis loops of the mesoporous NWs are shown in Figure 9. The main magnetic anisotropic features observed in the dense NWs are preserved: the Porous-A sample displays isotropic behavior with a magnetic easy-direction along the major-axis of the NWs, whereas an almost anisotropic behavior is found in the Porous-B sample.

However, the core-shell structure of the NWs, i.e., mesoporous core and dense shell, slightly affected the magnetic properties; see Table 2. Specifically, all curves of mesoporous NWs showed an increase of the coercive field, especially for the Porous-B sample, as well as more robustness of the magnetic disorder, which hindered a deep magnetic saturation up to 15 kOe. The χ_Hc_ values were comparable in the Dense-A and Porous-A NW arrays, whereas an evident decrease was found in Porous-B sample with respect to the Dense-B one.

All these magnetic features suggested that the formation of the mesoporous structure broadened the local magnetic anisotropy values, which influenced the overall magnetization process distributing the irreversible processes within a larger portion of the hysteresis loop and hindering the domain wall motion. This effect was mainly evident in the Porous-B sample.

In both Figure 8 and Figure 9, the hysteresis loops of the Dense-A (or porous A) sample show a distinct anisotropic behavior, with an easy axis preferably oriented parallel to the NWs’ long axis. Conversely, the hysteresis loops of sample B measured parallel and perpendicular to the NWs’ axis, almost coincide, indicating no clear anisotropy. This dissimilar behavior between both samples is mainly attributed to the different average interwire distance between the two samples (i.e., D = 390 nm for Dense-A and D = 270 nm for Dense-B). More specifically, the overall magnetic behavior of the NWs depends on the energy balance between three contributions: shape anisotropy, the dipolar field created between neighboring NWs, and the magnetocrystalline anisotropy. For an array of ordered NWs, the resulting effective anisotropy field can be expressed as [50,51,52,53,54,55]:

(1) $$H_{K,eff}=(N_{⊥}-N_{\left|\right|})M_{S}-\frac{6.3M_{S}\pi^{}r^{2}L}{D^{3}}+H_{mc}$$

For a two-dimensional array of cylindrical objects with radius r, length L, aspect ratio A_r_ = L/2r, and separation (i.e., interwire distance) D, the total dipolar field when all NWs are oriented along their long axis can be expressed as [51,52,53]:

(2) $$H_{dip,}{}_{0º}=\frac{4.2M_{S}\pi^{}r^{2}L}{D^{3}}$$

If all moments are aligned perpendicular to the NWs direction, then the dipolar field becomes:

(3) $$H_{dip,}{}_{90º}=\frac{-2.1M_{S}\pi^{}r^{2}L}{D^{3}}$$

Thus, the self-demagnetizing field of one NW can be expressed as:

(4) $$H_{demag}=(N_{⊥}-N_{\left|\right|})M_{S}$$

where N_║_ and N_⊥_ are the demagnetizing factors along the NW axis and its perpendicular direction and can be calculated as [54]:

(5) $$N_{\left|\right|}=\frac{4\pi }{A_{r}^{2}-1}\left[\frac{A_{r}}{2\sqrt{\left(A_{r}^{2}-1\right)}}\ln\left(A_{r}+\sqrt{A_{r}^{2}-1}\right)-1\right]$$

(6) $$N_{⊥}=\frac{4\pi -N_{\left|\right|}}{2}$$

Finally, H_mc_ is the magnetocrystalline anisotropy contribution.

In a first approximation, due to the shape anisotropy, an isolated NW with a large aspect ratio should display a square hysteresis loop when measured with a magnetic field applied parallel to the NW long axis, and a tilted (i.e., hard-axis loop) along the perpendicular direction, thereby behaving as a dipole with a clear anisotropic behavior. The other extreme would be when the NWs would be placed almost in direct contact with each other, thus resembling a continuous ferromagnetic film where, due to the shape anisotropy, the easy axis direction would be parallel to the film plane (i.e., perpendicular to the NW axis). In other words, interwire dipolar interactions can induce a crossover of the easy axis, from being parallel to the NWs’ long axis to being perpendicular to them. Such crossover in the magnetic easy axis has been observed in the literature in arrays of Co or Fe NWs [50,56].

In our case, since the interwire distance in the Dense/Porous-A sample was larger, the magnetic easy axis was still parallel to the NWs’ long axis. However, in the Dense/Porous-B sample, where the interwire distance was smaller, the easy axis direction became less defined and the NWs exhibited sheared hysteresis loops along both directions of measurement, thus suggesting that the magnetic easy axis was neither parallel nor perpendicular to the NW axis.

Examination of Equation (1) predicts that if H_K,eff_ is negative the magnetic easy axis will be perpendicular to the NWs axis; otherwise, for positive H_K,eff_ values, the magnetic easy axis will be in the parallel-to-NWs direction [52]. In a first approximation, if one neglects H_mc_ (which would be a plausible assumption for the case of polycrystalline Fe [50]), and if one assumes representative values of r and L for the herein investigated NWs, the values of D that would lead to an overall zero H_K,eff_ (i.e., D_cr_) would be in the range between 420 and 470 nm. Larger values of D would result in the NWs exhibiting clear anisotropy along the NW axis, whereas lower values of D would result in effective magnetic anisotropy along the perpendicular direction. This critical value of D that would lead to the crossover behavior was not far from the experimental D values in our work. Yet, our interwire separation was smaller than D_cr_ for both samples. This suggests that H_mc_ should possibly be taken into account for a more quantitative description of our observations. Adding a positive H_mc_ term in Equation (1) would result in a lower D_cr_, thus improving the agreement between this simple model and the obtained experimental results. In addition, strictly speaking, Equations (2)–(6) are valid only for ordered square arrays of cylindrical dots [51]. In our case, the NWs are disposed randomly inside the pores of the AAO templates, which has been reported to cause a tilt of the hysteresis loops and an increase of the saturation field [55]. Finally, magnetic curling effects cannot be completely disregarded since the diameter of the NWs is relatively large, and they are relatively long. Such inhomogeneous magnetization reversal configurations tend to decrease the dipolar field and the coercivity, as compared to coherent rotation in single-domain states [56].

### 3.4. SERS Studies

To compare the influence of chemical composition and morphology on the SERS enhancement, two different samples were taken into account. The first being Pd-rich Porous-A, fabricated in this study and held against a nanoporous FePd thin film that displayed notable SERS activity, as previously reported by Cialone et al. [57]. The latter sample, obtained by chemically dealloying a sputtered thin film, had a composition of Fe_60_Pd_40_, porous morphology, with a roughness of (3.4 ± 0.3) nm measured by atomic force microscopy and a bcc α(Fe,Pd) phase (XRD pattern provided in Figure S3 of the Supplementary File).

**Figure 10:**
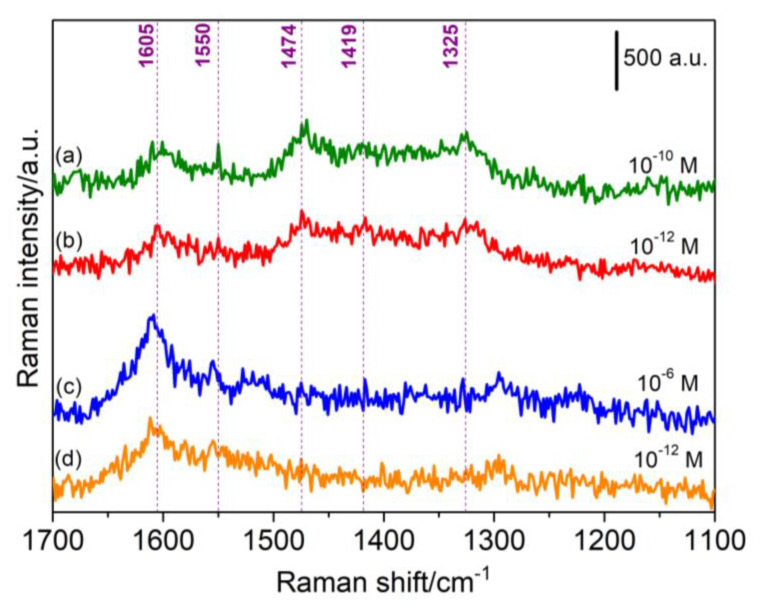
(**a**,**b**) SERS spectra shown by Porous-A NWs for bipy concentration of 10^−10^ M and 10^−12^ M respectively. (**c**,**d**) SERS spectra shown by nanoporous FePd thin film for bipy concentration of 10^−6^ M and 10^−12^ M respectively.

The SERS measurements were performed using Porous-A as a substrate for the detection of 4,4′-bipyridine (bipy) probe molecule using the same conditions adopted for the nanoporous FePd thin film. Figure 10a,b represent the spectra obtained for Porous-A with bipy concentration of 10^−10^ M and 10^−12^ M respectively. The sample shows well-enhanced signals with representative bipyridine peaks at 1605 cm^−1^, 1550 cm^−1,^ 1474 cm^−1^, 1419 cm^−1^, and 1325 cm^−1^. Accordingly, we witnessed a minor difference in signal intensities between the two spectra, approximately around 75 a.u., given the low concentrations of bipy [58,59]. A remarkably low detection limit of 10^−12^ M was recorded. The reason behind such impressive results lies in the mesoporous morphology of Porous-A as well as the adjacent arrays of the NWs embedded in close proximity, which are exceedingly conducive to the electromagnetic enhancement effect and can be regarded as a facile area for the generation of hotspots (Figure S2 of the Supplementary File).

Regarding the SERS results acquired by Cialone et. al., Figure 10c,d display the spectra obtained by the nanoporous thin film for the same analyte, i.e., 4,4′-bipyridine at concentrations of 10^−6^ M and 10^−12^ M respectively. The signals are quite evidently enhanced and the detection limit, in this case, was also found to be 10^−12^ M. Similar to Porous-A, the SERS performance, in this case, can be accredited to the nanoporous morphology of the as-dealloyed thin film which facilitated the LSPR phenomenon, producing amplified SERS signals combined with palladium clusters present on the surface. Hence, Porous-A showed excellent SERS performance akin to Cialone et. al.’s nanoporous FePd thin film, making these mesoporous NWs a promising candidate as a SERS active substrate.

## 4. Conclusions

In conclusion, successful fabrication of dense and mesoporous FePd NWs with Pd content ranging from 45 to 60 at.% has been achieved. A template- and micelle-assisted pulsed potentiostatic electrodeposition method was adopted utilizing AAO and PC templates of variable pore sizes. For dense NWs, an FePd electrolyte was used, while for the mesoporous counterparts, this electrolyte additionally consisted of a block copolymer, P-123, acting as a micelle-forming surfactant. Single-phase fcc NWs were obtained displaying a range of lengths, from 3.1 µm to 7.1 µm. Upon applying progressively negative potentials, the NWs were seen to become richer in Fe content. The NWs obtained from the AAO membranes had the typical branched structure at the bottom while those obtained from PC membranes showed cigar-like shape ends, in addition to the main cylindrical morphology. A core-shell structure was noticed in the case of the mesoporous NWs—a porous internal volume and denser non-porous outer surface. This structure was deemed to have a slight influence on the magnetic properties of the mesoporous NWs. Overall, the samples exhibited a soft ferromagnetic behavior. The shapes of the hysteresis loops (when measured parallel and perpendicular to the NWs long axis) were shown to be influenced by the shape anisotropy and interwire dipolar interactions, which in turn depended on the NWs dimensions. Besides, mesoporous NWs acting as SERS substrates exhibited excellent SERS performance with a low detection limit of 10^−12^ M for 4,4′-bipyridine probe molecule. The mesoporosity and the adjacent embedding of the NWs provided an easy ground for the LSPR effect as well as hotspot generation. The SERS performance of these NWs was found to be comparable with that of a nanoporous FePd thin film in the literature with similar composition and morphology. Thus, these NWs can be potential candidates as active substrates for a wide range of SERS-based applications.

## Figures and Tables

**Table 1:** E_on_ values used for the growth of dense and mesoporous FePd NWs, the resulting chemical composition, template used, mean diameter, and maximum length.

NW Type	–E_on_ (V)	Composition	Template, Pore Size (nm)	Mean Diameter (nm)	Maximum Length (µm)
Dense	1.05	Fe_55_Pd_45_	PC, 50	135	4.6
	1.15	Fe_51_Pd_49_	PC, 50	113	4.7
		Fe_47_Pd_53_	AAO, 20	174	5.7
	1.20	Fe_52_Pd_48_	PC, 30	65	3.1
		Fe_55_Pd_45_	PC, 100	222	5.2
		Fe_50_Pd_50_ (Dense-A)	AAO, 20	157	4.7
		Fe_56_Pd_44_ (Dense-B)	AAO, 100	197	3.5
	1.30	Fe_60_Pd_40_	PC, 100	156	7.1
Mesoporous	1.20	Fe_40_Pd_60_ (Porous-A)	AAO, 20	168	3.2
		Fe_40_Pd_60_ (Porous-B)	AAO, 100	197	2.6

**Table 2:** Magnetic properties evaluated from hysteresis loops reported in Figure 8 and Figure 9. Saturation field (H_s_), coercive field (H_c_), magnetic susceptibility evaluated at the coercive field χ_Hc_, and the normalized magnetization remanence (M_r_/M_s_). The nomenclature for the NW arrays is the same as in Table 1.

Sample	H Direction	H_s_ (kOe)	H_c_ (Oe)	χ_Hc_ (Oe^−1^)	M_r_/M_s_
Dense-A	PA	≈5	115	8.4 × 10^−4^	1.0 × 10^−1^
	PE	≈8	107	5.1 × 10^−4^	5.6 × 10^−2^
Dense-B	PA	≈15	10	4.1 × 10^−3^	3.6 × 10^−2^
	PE	≈15	10	1.4 × 10^−3^	1.4 × 10^−2^
Porous-A	PA	>15	128	9.7 × 10^−4^	1.1 × 10^−1^
	PE	>15	140	4.4 × 10^−4^	8.5 × 10^−2^
Porous-B	PA	>15	62	3.9 × 10^−4^	2.0 × 10^−2^
	PE	>15	71	4.6 × 10^−4^	3.9 × 10^−2^

## Data Availability

Data will be made available on request.

## Supplementary Materials

The following supporting information can be downloaded at: https://www.mdpi.com/article/10.3390/nano13030403/s1, Figure S1: Pulsed potentiostatic electrodeposition scheme: during t_on_, the potential E_on_ is applied at the selected deposition sites; during t_off_, the electrodes are brought back to the initial open-circuit potential E_off_; Figure S2: SEM image of the top surface of Porous-A nanowires partially embedded in the AAO template; Figure S3: (a) XRD pattern of the as-deposited Porous-A embedded in the AAO template. (b) XRD pattern of Cialone et. al’s nanoporous (NP) FePd thin film.
