# Supplementary material for: Electrochemical Synthesis, Magnetic and Optical Characterisation of FePd Dense and Mesoporous Nanowires
Source: Nanomaterials (Basel). 2023 Jan 19;13(3):403. doi: 10.3390/nano13030403 (PMC9920478; doi:10.3390/nano13030403)
Supplement: Supplementary file 1 [file nanomaterials-13-00403-s001.zip › nanomaterials-2142320-supplementary.pdf]

nanomaterials

Supplementary Material

Electrochemical Synthesis, Magnetic and Optical Characterisation of FePd Dense and Mesoporous Nanowires

Deepti Raj [1], Gabriele Barrera [2], Federico Scaglione [1], Federica Celegato [2], Matteo Cialone [1,3], Marco Coïsson [2], Paola Tiberto [2], Jordi Sort [4,5], Paola Rizzi [1,*] and Eva Pellicer [4]

1 Dipartimento di Chimica e Centro Interdipartimentale NIS (Nanostructured Interfaces and Surfaces), Università di Torino, Via Pietro Giuria 7, 10125 Torino, Italy
2 Istituto Nazionale di Ricerca Metrologica (INRIM), Str. delle Cacce 91, 10135 Torino, Italy
3 Physics Department, University of Genova, Via Dodecaneso 33, 16146 Genova, Italy
4 Universitat Autònoma de Barcelona, Campus de la UAB, Bellaterra, 08193 Barcelona, Spain
5 Institució Catalana de Recerca i Estudis Avançats (ICREA), Pg. Lluís Companys 23, 08010 Barcelona, Spain
* Correspondence: paola.rizzi@unito.it

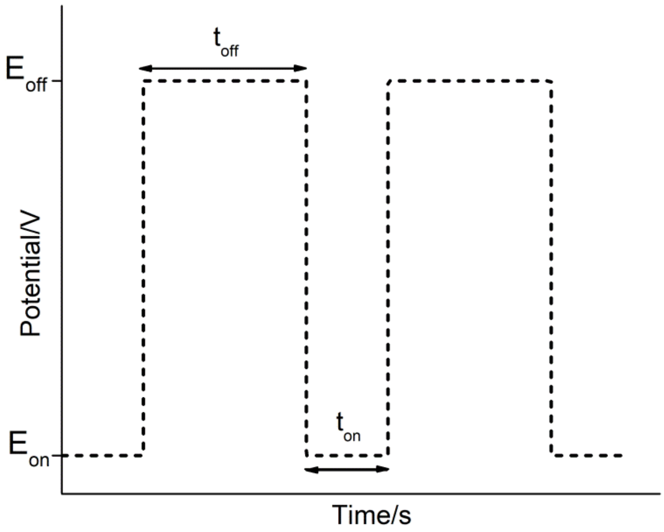

Figure S1. Pulsed potentiostatic electrodeposition scheme: during t_{on}, the potential E_{on} is applied at the selected deposition sites; during t_{off}, the electrodes are brought back to the initial open-circuit potential E_{off}.

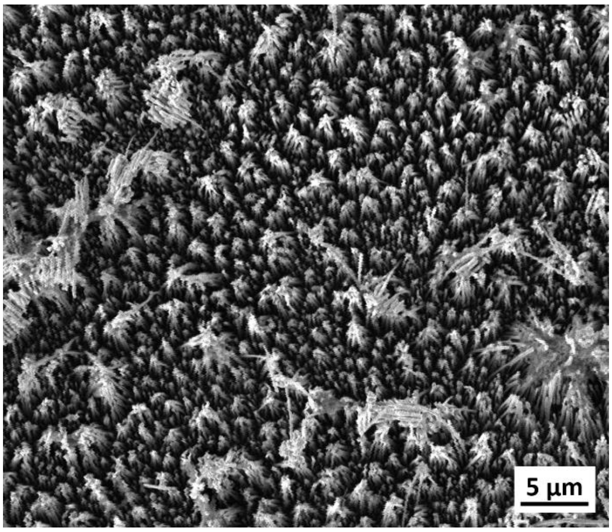

Figure S2. SEM image of the top surface of Porous-A nanowires partially embedded in the AAO template.

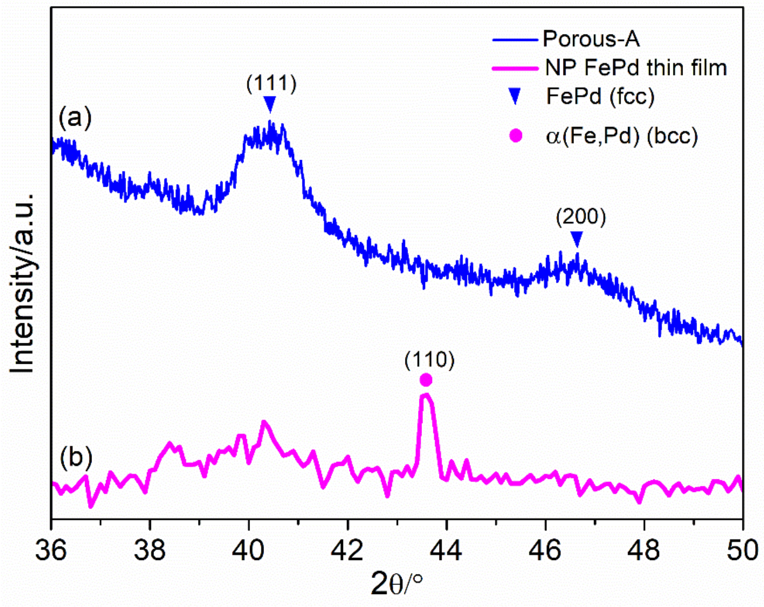

Figure S3. (a) XRD pattern of the as-deposited Porous-A embedded in the AAO template. **(b)** XRD pattern of Cialone et. al's nanoporous (NP) FePd thin film. .
